# Biofeedback Modalities Targeting Kyphosis and Lordosis: A Critical Appraisal Topic

**DOI:** 10.1155/bmri/3879174

**Published:** 2026-04-29

**Authors:** Reza Rajabi, Hooman Minoonejad, Fateme Khorramroo, Mostafa Jalili Bafrouei

**Affiliations:** ^1^ Department of Sport Injuries and Biomechanics, Faculty of Sport Sciences and Health, University of Tehran, Tehran, Iran, ut.ac.ir

**Keywords:** alignment, deformity, EMG, feedback, IMU, sagittal, sensor, spine, trunk

## Abstract

**Background:**

Postural deviations such as thoracic kyphosis and lumbar lordosis are prevalent consequences of sedentary behavior, contributing to pain and disability. We aim to review the evidence on biofeedback modalities as active, user‐engaged alternatives to passive correction targeting kyphosis and lordosis posture.

**Methods:**

A systematic search of PubMed, Web of Science, Scopus, and IEEE Xplore (inception: September 2025) identified studies on adult populations. Primary outcomes were spinal alignment measures; secondary outcomes included muscle activity, pain, usability, and satisfaction. The results of the included studies were synthesized in a qualitative narrative way.

**Results:**

Fourteen studies involving 288 participants met inclusion criteria. Most studies used accelerometers or IMUs for posture detection. The most frequently applied modality was vibration feedback (*n* = 6), followed by auditory (*n* = 3), visual (*n* = 3), and multimodal systems (*n* = 2). Across studies, short‐term improvements were consistent: reduced kyphotic (up to 8°) and lordotic (up to 10°) angles, decreased pain, and improved trunk posture (*p* < 0.05); enhanced awareness and spinal muscle relaxation (*p* < 0.05); and actuator‐based systems achieved up to 25° of active correction but lacked long‐term data. Modalities showed usability and user satisfaction and had postural measurement error of 1°–4° across studies.

**Conclusion:**

Biofeedback modalities—particularly vibration and auditory systems—demonstrate measurable improvements in spinal alignment and engagement. Evidence is limited by low methodological rigor, small sample sizes, and short‐term focus. Future research should investigate the effectiveness of biofeedback in longitudinal, real‐world trials, with multimodal feedback and ergonomic integration for sustained postural correction.

## 1. Introduction

The spine plays a fundamental role in supporting the human body, distributing loads, and maintaining balance during daily activities [1]. Correct spinal alignment is essential for musculoskeletal health, whereas poor posture can result in pain, disability, and impaired function [2, 3]. Among the most prevalent spinal postural deviations are kyphosis and lordosis, both of which involve abnormal curvature patterns. Kyphosis, characterized by excessive thoracic curvature, is often caused by poor sitting habits or muscle weakness and is associated with pain [4, 5], fatigue, and impaired respiratory function [6, 7] and gait [8, 9]. Lumbar lordotic curvature (LLC) has been widely debated in relation to low back pain (LBP): While excessive LLC may overload posterior structures, reduced LLC has also been linked with pain during prolonged sitting or lifting [10], particularly when combined with poor postural habits [11].

Modern lifestyles further reinforce these postural problems, as sedentary behavior, prolonged sitting during studying or office work, and frequent use of smartphones, laptops, and tablets exacerbate spinal malalignment [2, 3]. Over 3 billion smartphone users experience neck and shoulder discomfort associated with poor posture [2]. Office employees and students show high prevalence of slouched sitting and spinal curvature deviations, with kyphosis reported in up to 70% of computer users and 17%–63% of office workers reporting back or neck pain annually [12, 13]. The consequences of these conditions extend beyond physical discomfort, contributing to disability, reduced productivity, and high socioeconomic costs; back and neck pain are among the leading causes of global disability, with enormous healthcare expenditures and productivity losses [13].

In response, researchers have explored biofeedback‐based posture monitoring and correction systems as alternatives to prior physiotherapy‐based interventions [14] and passive devices such as ergonomic chairs or rigid braces, which often suffer from poor compliance or limited effectiveness [7, 13]. Biofeedback delivers real‐time information that enables users to actively adjust their posture, thereby helping prevent the development of postural abnormalities. Several modalities have been tested: vibration feedback alerts through haptic cues, auditory feedback uses alarms or tones, and visual feedback provides smartphone notifications or display outputs [13, 15]. These systems rely on diverse sensing technologies. Inertial measurement units (IMUs) and accelerometer–gyroscope combinations monitor angular deviations of the spine [2, 3]. Electromyography (EMG) assesses muscle activity patterns [10]. Flex sensors, textile‐based strain sensors, tensile sensors, inductive sensors, pressure sensors, and optical fibers have also been embedded into garments, belts, or orthoses to track postural angles [2, 6]. Although some prototypes have advanced to include actuators (e.g., DC motors, fluid‐driven devices, or exoskeleton braces) for active correction, many remain limited to monitoring and alerting functions [6, 12].

Despite substantial progress, several important gaps remain in the literature. First, the effectiveness of different biofeedback modalities across common daily activities, such as prolonged sitting, studying, device use, or lifting has not been systematically compared [10]. Second, most systems primarily focus on monitoring rather than active correction, and corrective prototypes are often bulky, uncomfortable, or impractical for sustained daily use [2, 13]. Third, evidence regarding long‐term adherence, clinical outcomes, and real‐world usability remains limited [7, 15]. Given these limitations, a comprehensive synthesis of current biofeedback approaches is needed. Therefore, this systematic review is aimed at critically avaluating and synthesizing the available evidence on biofeedback modalities targeting kyphosis and lordosis. Specifically, it examines (1) the types of feedback provided, (2) the sensing technologies employed, and (3) their effectiveness and practical applicability of these systems in preventing or correcting spinal deviations including kyphosis and lordosis. This work seeks to consolidate current findings, identify limitations, and inform future development of posture‐correction systems for both clinical and everyday applications.

## 2. Materials and Methods

### 2.1. Study Design

This study was conducted as a systematic review following the Preferred Reporting Items for Systematic Reviews and Meta‐Analyses (PRISMA) guidelines [16]. The objective was to identify, evaluate, and synthesize studies examining the usability and effects of biofeedback‐based interventions on biomechanics and postural alignments of individuals with kyphosis and lordosis. The protocol was registered on Prospero (CRD420261296160).

### 2.2. Search Strategy

We searched PubMed, Web of Science, Scopus, and IEEE Xplore up to September 30, 2025. The databases were interrogated with keywords following the PICOT framework:

This review focused on adults, either healthy or with kyphosis and lordosis, studied in laboratory, clinical, or real‐world settings. Interventions included any biofeedback modality targeting kyphosis and lordosis, such as visual, auditory, or haptic feedback, wearable sensors or garments, and virtual or augmented reality systems. Comparators included standard care, educational interventions, no feedback, or alternative feedback approaches. Primary outcomes were objective spinal posture measures (e.g., thoracic kyphosis, lumbar lordosis, and trunk flexion). Secondary outcomes included muscle activity, pain or discomfort, device usability, and user satisfaction. All intervention durations and follow‐up periods were eligible. The final search strategy included two domains:

1. Biofeedback, feedback.
2. Spin∗ OR thoracic OR lordosis OR kyphosis OR lumbar.
3. (#1 AND #2).

To ensure completeness, reference lists of relevant studies were manually screened.

### 2.3. Eligibility Criteria

Studies were included based on the following PICO criteria:

• Population: healthy adults or adults with kyphosis or lordosis problems without restrictions on age, sex, or ethnicity.
• Intervention: biofeedback modalities for kyphosis and lordosis (visual, auditory, haptic, and wearable) in laboratory, clinical, or real‐world settings.
• Comparators: standard care, education, no feedback, or alternative biofeedback.
• Outcomes: thoracic kyphosis, lumbar lordosis, muscle activity (EMG), pain/discomfort, usability, and satisfaction.

Study design include randomized controlled trials, cross‐sectional studies, prototype validation, and usability studies.

Language and publication status comprise English studies.

Exclusion criteria were nonbiofeedback studies, those lacking kyphosis and lordosis outcomes, reviews or conference studies, or studies without an English abstract.

### 2.4. Study Selection

The screening of titles, abstracts, and full texts was performed independently by F.K. and M.J.B., following the predefined inclusion criteria. Any disagreements were resolved through discussion until a consensus was reached.

### 2.5. Quality Assessment

The methodological quality of the included studies was appraised using standardized tools appropriate to each study design. Validity and reliability studies were evaluated with the COSMIN checklist [17], whereas prototype testing studies were assessed using the JBI Critical Appraisal Checklist for Text and Opinion Papers [18]. All cross‐sectional and quasi‐experimental studies were appraised with the JBI Cross‐Sectional Checklist [19]. Quality assessment was performed independently by two reviewers, with agreement quantified using Cohen′s *κ* (target: ≥ 0.75). Any disagreements were resolved through discussion to ensure accuracy and consistency.

### 2.6. Data Collection

Data extraction from the included studies was performed by F.K. and verified by M.J.B., with additional discussion performed for any discrepancies prior to final dataset consolidation. Extracted information included study inclusion criteria, participant characteristics, funding sources, and the biofeedback types and tools used.

### 2.7. Synthesis of Results

The data from the included studies were synthesized using a qualitative narrative approach to integrate findings across different biofeedback modalities targeting kyphosis and lordosis. Results were extracted from each study focusing on key outcome measures, including changes in spinal angles (e.g., trunk deviation, lumbar lordotic angle (LLA), and thoracic kyphosis), muscle activity (e.g., EMG of cervical erector spinae, trapezius, and lumbar muscles), and posture‐correction outcomes (e.g., percentage improvement or angular reduction). Studies were categorized according to the type of feedback provided, vibratory, auditory, tactile, visual, and fluid‐driven systems and summarized descriptively, as substantial heterogeneity existed in study designs, measurement techniques and outcome reporting. Additional qualitative outcomes, including user experience and device usability, were also considered to evaluate the practical applicability of systems. Where applicable, findings were compared with nonbiofeedback posture‐correction approaches reported in the included studies.

## 3. Results

### 3.1. Study Selection

**Figure 1:**
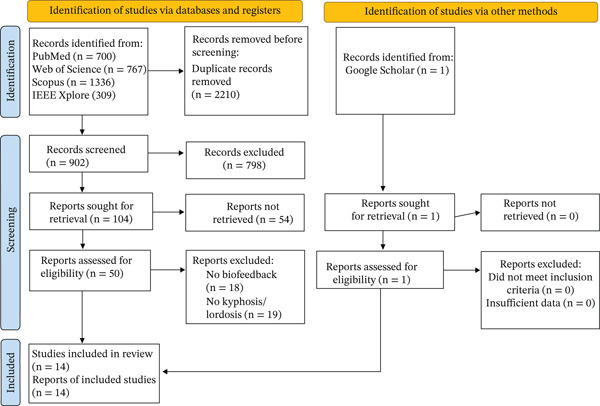
Flow diagram of searching and screening studies.

The initial literature search yielded a total of 3112 records, including 700 from PubMed, 767 from Web of Science, 1336 from Scopus, and 309 from IEEE Xplore, with 902 unique studies after removing duplicates. Following title, abstract, and full‐text reviews, 13 studies were retained. One additional study was identified through manual searching on Google Scholar, resulting in a final inclusion of 14 studies [2, 6, 10, 13, 15, 20–28]. Figure 1 depicts the selection process and the number of studies excluded at each step.

### 3.2. Study Characteristics

Fourteen studies with 288 participants published between 2006 and 2025 investigated biofeedback interventions for managing kyphosis and lordosis (excessive thoracic kyphosis and/or lumbar lordosis) in healthy or kyphotic or lorditic individuals, statically or dynamically, using wearable or seat‐based systems during activities such as computer typing, prolonged sitting, or daily movements. Devices incorporated a range of sensing technologies, including accelerometers (*n* = 5), IMUs (*n* = 4), smart garments with integrated sensors (*n* = 2), 3D motion sensors (*n* = 1), flex sensors (*n* = 1), and EMG (*n* = 1). Feedback modalities varied: vibration was the most common (*n* = 6), followed by auditory (*n* = 3), visual (*n* = 3), manual/verbal (*n* = 1), and multimodal systems combining more than one type of feedback (*n* = 2).

Sample sizes ranged from single‐case designs to small groups of 30 participants, with a median of around 10–15 individuals. Populations studied included healthy young adults, kyphosis, and LBP. However, the outcomes of interests targeted kyphosis or lordosis. Reported outcomes were diverse, spanning angular measures of spinal posture (kyphotic angle, lumbar lordosis, and cervical flexion/extension), trunk stability, EMG‐derived muscle activity, self‐reported pain, and device usability or wearability metrics.

Methodologically, cross‐sectional designs predominated (*n* = 8), alongside validity or reliability studies (*n* = 2), quasi‐experimental interventions (*n* = 2), prototype evaluations (*n* = 1), and a single randomized controlled trial (*n* = 1).

Investigated outcomes included: head tilt, forward head/shoulder, neck/trunk flexion, spinal angles (cervical, thoracic, lumbar, pelvic, thoracolumbar, and lordotic/kyphotic—static and dynamic), spinal motion/stability, EMG (lower/upper limbs, erector spinae, trapezius, and obliques during gait/typing), pressure from load, pain (VAS and LBP), and disability (Oswestry). Detailed individual study findings are summarized in Table 1.

**Table 1:** Study characteristics of included studies.

Author and year	Sensor	Feedback	Participants	Task	Outcome	Study design	Funding or conflict of interests
Kuo et al. 2021[13]	Accelerometer	Vibration	21 healthy young	Computer typing	Head tilt, neck flexion, upper cervical, lower cervical, thoracic, lumbar, and pelvic plane	One‐group quasi‐experimental study	Ministry of Science and Technology of Taiwan (R.O.C.) (Grant Number: MOST 106‐2410‐H‐006‐083‐MY2) and the National Cheng Kung University Hospital, Tainan, Taiwan (R.O.C.) (Grant Number: NCKUH‐10506017).
Claus et al. 2009[24]	3D motion sensors	Manual and verbal	Lordosis and slumped posture, 10 males with a mean (SD) age of 23 (9) years, height of 178 (10) cm, and weight of 75 (9) kg	Sitting	Thoracic, lumbar, and thoracolumbar angles	Cross‐sectional	NA
Cortell‐Tormo et al. 2019[25]	Inertial textile	Visual	15 Healthy volunteers (10 men and 5 women)	Prone	Lumbar stability	Validity	NA
Kim et al. 2025[10]	Inertial	Auditory	10 Males LBP	Repeated flexion task (pick‐up and squatting, sitting, lifting, and mopping)	Lumbar lordotic angle	Cross‐sectional	Bio and Medical Technology Development Programs of the National Research Foundation (NRF), funded by Ministry of Science and ICT, Republic of Korea (Grant Number: 2016M3A9F1941984); and by the Translational Research Program for Rehabilitation Robots, funded by the National Rehabilitation Center, Ministry of Health and Welfare, Republic of Korea (Grant Number: NRCTR‐EX24002).
Lou et al. 2012[28]	IMU Garment	Vibration	One Kyphotic and four healthy controls	Sitting	Kyphotic angle	Cross‐sectional	NA
Ijaz et al. 2023[6]	Imu and flex	Force	Kyphosis	Sitting	Posture	Cross‐sectional	The Ministry of Education and Science (MES), Republic of Kazakhstan, Grant No. SEDS2022014, the source of funding code is 055.01.01 and, by Nazarvayev University under the Faculty Development Competitive Research Grant Program (FCDRGP) Grant No. 021220FD1551
Asadullah et al. 2022[2]	Flex and IMU	Vibration	Kyphosis	Standing	Pressure made by applied load	Prototype testing	JSPS Grant‐in‐Aids for Scientific Research (C) 21K03930.
MohammadZadeh et al. 2019[20]	Bending sensor brace	Vibration	17 kyphotic students aged between 18 and 30 years	Standing	Pressure made by applied load	Validity and reliability	Ministry of Education and Science (MES), Republic of Kazakhstan, Grant No. SEDS2022014, the source of funding code is 055.01.01 and, by Nazarvayev University under the Faculty Development Competitive Research Grant Program (FCDRGP) Grant No. 021220FD1551.
Piran Hamlabadi et al. 2024[27]	Bending sensor brace	Vibration	15 Kyphotic males	Walking	EMG lower extremity during gait	Cross‐sectional	NA
Piran Hamlabadi and Akbarigharibe 2024[27]	Bending sensor brace	Vibration	30 kyphotic male employees	Typing	EMG upper extremity during typing	Cross‐sectional	NA
Park et al. 2016[21]	Contact feedback device (pressure)	Vibration	18 Kyphotic students	Typing	EMG thorqcic erector spinae, VAS and Kyphosis angle	Cross‐sectiona	National Research Foundation of Korea Grant funded by the Korean Government (NRF‐2013S1A5B8A01055336)
Kim et al. 2023[22]	EMG	Visual and auditory	20 healthy young adults (11 males and 9 females) who worked with a computer for at least 4 h a day and 5 days a week	Typing	Erector spinae and upper trapezius EMG and neck angle	Cross‐sectional	Brain Korea 21FOUR Project, Korean Research Foundation for the Department of Physical Therapy in the Graduate School of Yonsei University (grant number 2021‐51‐0151): and Basic Science Research Program through the National Research Foundation of Korea (NRF), funded by the Ministry of Education (Grant Number: 2021R1F1A104792912)
Rahimi et al. 2020[15]	Trainer	Touch	20 Kyphotic	Standing	Kyphotic angle	Cross‐sectional	NA
Wong et al. 2008[23]	Inertial sensors	Auditory	Five healthy subject	Sitting and standing	Thoracic and lumbar sagittal and frontal planes	Prototype testing	NA

### 3.3. Quality Assessment

Table 2 summarizes the risk of bias and methodological quality of the included studies according to their study design, appraisal tools, and specific assessment criteria. Overall, methodological quality varied across studies.

**Table 2:** Results of risk of bias and quality assessment.

**Study ID**	**Study design and appraisal tool**	**Clear inclusion criteria**	**Detailed subject and setting**	**Valid and reliable exposure**	**Standard measurement**	**Confounding factors identified**	**Sterategies to deal with confounding factors state**	**Valid and reliable measurement**	**Appropriate statistical measurement**
Kuo et al. 2021[13]	One‐group quasi‐experimental study: JBI Cross‐Sectional Checklist	Yes	Yes	Yes	Yes	Yes	Unclear	Yes	Yes
Claus et al. 2009[24]	Cross‐sectional: JBI Cross‐Sectional Checklist	Yes	Yes	Yes	Yes	Yes	Unclear	Yes	Yes
Kim et al. 2025[10]	Cross‐sectional: JBI Cross‐Sectional Checklist	Yes	Yes	Yes	Yes	Yes	Unclear	Yes	Yes
Piran Hamlabadi et al. 2024[27]	Cross‐sectional: JBI Cross‐Sectional Checklist	Yes	Yes	Yes	Yes	Yes	Unclear	Yes	Yes
Piran Hamlabadi and Akbarigharibe 2024[27]	Cross‐sectional: JBI Cross‐Sectional Checklist	Yes	Yes	Yes	Yes	Yes	Unclear	Yes	Yes
Park et al. 2016[21]	Cross‐sectional: JBI Cross‐Sectional Checklist	Yes	Yes	Yes	Yes	Yes	Yes	Yes	Yes
Kim et al. 2023[22]	Cross‐sectional: JBI Cross‐Sectional Checklist	Yes	Yes	Yes	Yes	Yes	Unclear	Yes	Yes
Rahimi et al. 2020[15]	Cross‐sectional: JBI Cross‐Sectional Checklist	Yes	Yes	Yes	Yes	No	No	Yes	Yes
Lou et al. 2012[28]	Cross‐sectional: JBI Cross‐Sectional Checklist	Yes	Yes	Yes	Yes	No	No	Yes	Yes
Study ID	**Study design and appraisal tool**	**Percentage of missing items given**	**Description of how missing items were handled**	**Sample size included in the analysis adequate**	**Gold** **standard**	**Any important flaws in the design or methods of the study**	**Correlations or the area under the receiver operating curve calculated**	**Sensitivity and specificity determined**	
Cortell‐Tormo et al. 2019[25]	Validity: COSMIN (BOX H)	No	No	No	No	Yes	Partially	No	
MohammadZadeh et al. 2019[20]	Validity: COSMIN (BOX H)	No	No	No	Yes	Yes	No	Yes	
Study ID	**Study design and appraisal tool**	**Identified source of opinion**	**Researchers experties**	**Focus on population interests**	**Logicly analysed**	**Reference to litterature**	**Incongruence with litterature**		
Asadullah et al. 2022[2]	Prototype testing: JBI text and opinion papers	Yes	Yes	Yes	Yes	Yes	Unclear		
Wong et al. 2008[23]	Prototype testing: JBI text and opinion papers	Yes	Yes	Yes	Yes	Yes	Unclear		
Ijaz et al. 2023[6]	Prototype testing: JBI text and opinion papers	Yes	Yes	Yes	Yes	Yes	Unclear		

Two studies evaluating measurement validity (e.g., Cortell‐Tormo et al. [25] and MohammadZadeh et al. [20]) were assessed using the COSMIN checklist. Both studies scored “No” across several criteria, including sample description, sample size justification, and comparison with a gold standard, indicating limited methodological rigor.

Prototype testing studies (Asadullah et al. [2], Ijaz et al. [6], and Wong et al. [23]), assessed using the JBI text and opinion appraisal tool, demonstrated mixed results. Although these studies generally identified their information sources and reported relevant expertise, several criteria—such as consistency with existing literature—were rated as “unclear.”

Most of the included studies employed cross‐sectional designs and were assessed using the JBI Cross‐Sectional Checklist. These studies generally demonstrated stronger methodological quality, with “yes” ratings for key criteria such as clearly defined inclusion criteria, detailed participant descriptions, valid and reliable exposure measurements, and appropriate statistical analysis. However, confounding factors and strategies to address them were frequently rated as “unclear” or “no,” with the exception of Park et al. 2016 [21], which explicitly addressed potential confounders.

Overall, cross‐sectional studies tended to demonstrate higher methodological quality, whereas prototype‐testing and validity/reliability studies showed greater methodological limitations, particularly regarding sample size justification, confounding control, and methodological transparency.

### 3.4. Sensor Modalities and Measurement Accuracy

Most studies demonstrated high accuracy in spinal angle detection:

Cortell‐Tormo et al. [25] and Wong et al. [23] reported measurement errors below 3° when compared with motion analysis systems.

Lou et al. [28] and Kuo et al. [13] validated IMU‐based garments and accelerometer feedback with mean angular errors between 2°–4°, confirming reliability for static and dynamic posture assessments.

### 3.5. Feedback Modalities and Postural Outcomes

#### 3.5.1. Vibratory Feedback

Vibration was the most common modality [13, 20, 21, 26–28].

Kuo et al. [13] reported significant reductions in neck flexion (*p* < 0.001), thoracic kyphosis (*p* = 0.033), and pelvic tilt (*p* = 0.021) using accelerometer‐driven vibration feedback.

Lou et al. [28] demonstrated short‐term improvements, with kyphotic angle reductions of 8^°^ ± 2^°^ after repeated feedback sessions.

Park et al. [21] found that a contact pressure feedback device significantly reduced pain scores and thoracic kyphosis angle (*p* < 0.05).

Hamlabadi et al. ([26, 27]) showed EMG modulation during gait and typing tasks, indicating altered muscle‐recruitment patterns with brace‐integrated vibratory feedback.

#### 3.5.2. Auditory Feedback

Kim et al. [10] found auditory feedback improved LLA during sitting and mopping tasks by 10.2°–10.5° (*p* < 0.001) but had a limited effect during squatting.

Wong et al. [23] observed postural improvement in trunk curvature when auditory feedback was provided during daily activities, with a mean error of < 3.1° versus motion capture reference.

#### 3.5.3. Visual Feedback

Cortell‐Tormo et al. [25] reported strong user acceptance and < 1° measurement error in static posture tasks.

Kim et al. [22] showed significant reductions in neck angle (*p* = 0.014) and cervical erector spinae EMG activity (*p* = 0.008) with combined visual and auditory PC feedback, supporting its neuromuscular benefit during computer work.

#### 3.5.4. Tactile and Force‐Based Feedback

Rahimi et al. [15] demonstrated that tactile feedback combined with corrective exercise led to a greater kyphosis reduction (−12%) than exercise alone (−8%) (*p* = 0.018).

Ijaz et al. [6] and Asadullah et al. [2] used pneumatic or McKibben actuator‐based systems, reporting postural correction forces of 19–34 N and angle reduction up to 25°, confirming feasibility for active mechanical correction.

#### 3.5.5. Manual and Verbal Feedback

Claus et al. [24] confirmed that manual facilitation was necessary to help participants differentiate between thoracolumbar and lumbar curvature adjustments, highlighting the limited efficacy of visual imitation alone.

### 3.6. Overall Efficacy

Across modalities, vibration and auditory feedback produced the most consistent short‐term postural corrections. Visual feedback enhanced awareness and muscle relaxation but required user engagement. Force‐ or actuator‐based systems provided measurable correction but lacked long‐term validation. Several studies (e.g., [10, 15, 21]) demonstrated statistically significant improvements (*p* < 0.05) in spinal angle correction or pain reduction, whereas others focused primarily on device validation rather than clinical outcomes.

### 3.7. Comparison of Biofeedback‐Based Approaches and Traditional Methods for Kyphosis and Lordosis Correction and Its Mechanisms

Traditional posture‐correction methods for kyphosis and lordosis, such as ergonomic education [29, 30], bracing [31], manual cueing [32], and corrective exercises, rely on passive support or intermittent supervision, yielding improvements mainly during conscious attention or physical constraint but with limited long‐term retention. In contrast, biofeedback‐based approaches deliver continuous real‐time feedback, improving proprioceptive accuracy [33], consistent muscle engagement [34], and motor learning without requiring human intervention, physical contact, embarrassment, or scheduled sessions, enabling 24/7 autonomous use that translates effectively to real‐world activities. This self‐directed nature empowers users to achieve sustained, independent postural regulation far beyond the constraints of traditional methods [35]. Biofeedback systems provide real‐time cues that increase postural awareness, allowing users to consciously activate stabilizing muscles and reduce maladaptive movement patterns. Over time, consistent feedback strengthens cortical representations of upright alignment and promotes automatic postural control without external cues [36].

In theory, posture correction relies on sensorimotor retraining [33] that engages neuroplasticity, the brain′s ability to reorganize neural pathways in response to repeated, meaningful input [37]. Preventive effects emerge as these newly reinforced motor patterns become habitual, reducing the likelihood of returning to slouched or hyperlordotic positions during daily tasks [38]. As a result, motor cortex activity increases by 30% [39], promoting postural stability [40].

### 3.8. User Experience and Practicality

Participants rated most wearable systems as comfortable and usable [20, 25], though long‐term adherence data were scarce. Only Lou et al. [28] and Wong and Wong [23] examined extended wear, showing tolerance for 2–3 h of daily use.

## 4. Discussion

The limited findings from this critical appraisal underscore the promising role of biofeedback modalities in addressing thoracic kyphosis and lumbar lordosis, particularly in the context of sedentary lifestyles and prolonged device use. Across the 14 included studies, with 288 participants, biofeedback systems consistently demonstrated short‐term improvements in spinal alignment, with reductions in kyphotic and lordotic angles ranging from 8° to 25° depending on the modality and task. Vibration feedback emerged as the most frequently employed and effective approach, yielding statistically significant enhancements in neck flexion, thoracic kyphosis, and pelvic tilt during activities like computer work and sitting [13, 21, 26–28]. Auditory feedback, although less common, showed comparable efficacy in dynamic tasks such as mopping, where it facilitated LLA corrections of over 10° [10, 23], potentially due to its ability to integrate seamlessly into auditory environments without physical encumbrance. The included studies had small sample sizes, assessed acute effects, and varied regarding study design, feedback type, measurement tools, making us unable to synthesize data and retrieve strong conclusions.

Visual feedback modalities, often delivered via smartphone apps or displays in short‐term,

were particularly effective in fostering postural awareness and reducing muscle activity, as evidenced by decreased cervical erector spinae EMG signals during computer tasks [22, 25]. These systems encourage self‐monitoring and neuromuscular relaxation, which may explain their superiority in static postures compared with more intrusive haptic methods. However, multimodal combinations, integrating vibration with auditory or visual elements, offered additive benefits, such as improved trunk stability and reduced pain scores, suggesting synergistic effects that warrant further exploration [2, 6, 15]. In contrast, actuator‐based systems, including pneumatic and McKibben‐driven devices, provided active mechanical correction with forces up to 34 N, achieving substantial angle reductions but at the cost of bulkiness and limited portability [2, 6]. This highlights a tradeoff between passive alerting and active intervention, where the former excels in usability for daily activities, whereas the latter may be better suited for therapeutic settings requiring enforced alignment.

Sensing technologies played a pivotal role in the accuracy and applicability of these systems. Accelerometers and IMUs dominated the reviewed studies, offering reliable detection of angular deviations with errors typically below 3°–4° when validated against gold‐standard motion capture [13, 23, 25, 28]. These inertial sensors are advantageous for their low cost, wireless capabilities, and suitability for wearable integration, enabling monitoring during dynamic activities like gait or typing [26, 27]. EMG‐based approaches added value by capturing muscle recruitment patterns, revealing how biofeedback modulates erector spinae and trapezius activity to prevent fatigue [10, 22]. Emerging alternatives, such as flex sensors in smart garments, showed promise for unobtrusive, garment‐embedded tracking but were underrepresented, appearing in only two studies [2, 6]. This sensor diversity reflects an evolution from rigid, lab‐based systems to flexible, IoT‐enabled wearables, yet inconsistencies in multiplanar detection, often limited to sagittal plane measurements, underscore the need for advanced sensor fusion to capture rotational or lateral deviations more comprehensively.

Comparatively, biofeedback through virtual reality or wearable sensors [25, 41] outperforms passive interventions like ergonomic chairs or braces, which rely on user compliance without real‐time guidance and often fail to sustain corrections beyond initial use [7]. The active engagement fostered by biofeedback aligns with motor learning principles, promoting habituation through repeated cues and potentially leading to internalized postural improvements [42]. However, the heterogeneity in study designs, predominantly cross‐sectional with small samples, precludes definitive conclusions on comparative superiority across modalities. For instance, while vibration reduced kyphosis more effectively than visual cues in prolonged sitting [13, 21], auditory feedback showed advantages in task‐specific scenarios [10], suggesting context‐dependent efficacy. A recent study [43] showed that auditory biofeedback and external focus of attention compared with internal focus can lead to more improvements. This review collects studies revealing that although short‐term biomechanical outcomes are robust, evidence on behavioral persistence and integration into occupational or rehabilitative routines remains sparse.

These results have broader implications for musculoskeletal health in an era of increasing digital dependency. By targeting preventable deviations like slouched sitting, biofeedback could mitigate the socioeconomic burden of back and neck pain, which accounts for billions in annual healthcare costs [13]. Integration with emerging technologies, such as augmented reality or AI‐driven personalization, could further enhance adaptability, allowing systems to adjust feedback thresholds based on user fatigue or activity type. Nonetheless, the predominance of healthy young adult samples limits applicability to clinical populations with chronic kyphosis or lordosis, where comorbidities like osteoporosis or disc degeneration may alter responsiveness.

## 5. Clinical Bottom Line

Biofeedback modalities, like the Lumo Lift, smart vest, and fluid‐driven correctors, offer effective short‐term, real‐time solutions for correcting spinal kyphosis and lordosis and reducing associated musculoskeletal discomfort during dynamic and static daily activities. By integrating advanced sensors and mobile applications, these systems enhance user engagement and precision. Clinicians should consider patient context and feedback preferences when selecting modalities to optimize outcomes.

## 6. Limitations of Included Studies

Most studies reviewed faced small, homogeneous samples and short intervention durations, limiting generalizability to broader populations or patients with postural disorders [10, 22, 26]. Many assessed only immediate or short‐term effects, leaving long‐term adherence and behavior change unclear [13, 21]. Methodological issues included static rather than time‐series data [22], nonrandomized orders and lack of crossover designs [10], and omission of discomfort or fatigue measures [27]. Technical challenges were frequent: inaccurate sensor placement [20], limited multiplanar or segmental motion detection [25], user “cheating” around feedback [44], and device constraints such as weight, power, and Wi‐Fi reliance [6]. Some participants also reported discomfort or distraction, especially with vibrotactile systems [13]. External factors like COVID‐19 further restricted sample sizes [15].

## 7. Limitations of the Review

Our study included only published English studies, and we did not search other languages or unpublished gray literature that may induce publication bias.

## 8. Future Directions

Future work should employ larger, diverse cohorts with longitudinal, real‐world trials to confirm clinical and ergonomic outcomes [10, 21, 22]. Stronger designs, including randomized crossovers and composite measures of discomfort, fatigue, and muscle activity, are needed [10, 27]. Technological priorities include accurate, real‐time multiplanar monitoring with wearable, user‐friendly designs [25], alternative sensors like infrared [44], and individualized feedback thresholds matched to activities [10]. Comparing haptic, visual, and auditory cues may reduce distraction at work [44]. Practical improvements—lighter materials, rechargeable batteries, faster processors, and integration with smartphones, smartwatches, or smart textiles—could enhance compliance [6, 25]. Furthermore, biofeedback interventions targeting kyphosis and lordosis should be evaluated under both static (e.g., prolonged sitting) and dynamic conditions (e.g., during gait and functional tasks [35]) to ensure comprehensive assessment of postural control across real‐world activities. Finally, collaboration with physical therapists remains essential for tailoring feedback and ensuring clinical relevance [15, 44].

## 9. Conclusion

Limited evidence by low methodological rigor, small sample sizes and short‐term focus demonstrated that biofeedback modalities, including vibratory, auditory, tactile, and visual systems, demonstrate significant potential in correcting spinal posture, surpassing passive methods like braces or lumbar supports. Innovations like fluid‐driven correctors and IoT‐integrated wearables offer proactive and user‐friendly solutions, though their efficacy varies by context and feedback type. By building on prior research, these modalities provide dynamic, precise interventions, making them valuable tools for posture correction in clinical and occupational settings. Future research should prioritize high quality and long‐term clinical, longitudinal, real‐world trials, multimodal feedback optimization, and ergonomic integration for sustained postural correction.

## Author Contributions

**R.R.**: conceptualization, formal analysis, investigation, project administration, supervision, validation, and writing—review & editing. **H.M.**: conceptualization, formal analysis, methodology, project administration, supervision, validation, and writing—review & editing. **F.K.**: conceptualization, data curation, formal analysis, investigation, methodology, project administration, resources, software, validation, visualization, writing—original draft, and writing—review & editing. **M.J.B.**: data curation, formal analysis, investigation, methodology, project administration, resources, software, validation, visualization, and writing—review & editing.

## Funding

No funding was received for this manuscript.

## Conflicts of Interest

The authors declare no conflicts of interest.

## Supporting information


**Supporting Information** Additional supporting information can be found online in the Supporting Information section. Figure S1: PRISMA 2020 checklist.

## Data Availability

All relevant data are included in the manuscript.
